# Corticosteroids in bacterial severe community-acquired pneumonia: lessons from recent trials

**DOI:** 10.3389/fmed.2025.1718570

**Published:** 2025-11-05

**Authors:** Thanh Luan Nguyen, Hoang Ngoc Thao Duong, Cong Dang Tran, Phuc Tuong Pham, Van Hoang Nam Ho, Van Phieu Duong

**Affiliations:** ^1^Faculty of Medicine, Nam Can Tho University, Can Tho, Vietnam; ^2^Critical Care Department, Nam Can Tho University Medical Center, Can Tho, Vietnam

**Keywords:** severe community-acquired pneumonia, corticosteroids, bacterial pathogens, inflammatory phenotypes, therapeutic regiments

## Introduction

Severe community-acquired pneumonia (SCAP) is a leading cause of intensive care unit (ICU) admission and mortality worldwide (1, 2). Bacterial SCAP, distinct from viral or fungal infections, often triggers a dysregulated host response with systemic inflammation, endothelial dysfunction, and organ injury (3), providing a rationale for adjunctive immunomodulatory therapies such as corticosteroids (3, 4).

Corticosteroids have been widely investigated in pneumonia and sepsis because of their potential to attenuate cytokine release, preserve vascular integrity, and limit lung injury (4). Although the latest guidelines suggest or conditionally recommend corticosteroids in SCAP (1, 5), evidence from randomized controlled trials (RCTs) remains inconsistent. Some studies demonstrated reductions in treatment failure or mortality (6–8), while others showed no significant clinical benefit (9, 10). These divergent findings likely reflect variability in enrolled populations, causative pathogens, corticosteroid regimens, and study designs (11, 12).

Emerging data suggest that corticosteroids effects depend on systemic inflammation and treatment protocol (13, 14), emphasizing the need for individualized rather than universal use. This paper revisits recent clinical trials of corticosteroids in SCAP, distills key lessons, examines challenges related to heterogeneous etiologies, inflammatory phenotypes, and therapeutic regimens, and outlines implications for current practice and future research.

## Rationale and biological basis of corticosteroid therapy

The pathophysiology of SCAP is marked by an excessive and dysregulated host immune response that contributes substantially to lung injury and systemic complications. During the acute phase, pathogen recognition leads to activation of nuclear factor κB (NF-κB) and other transcriptional pathways, with subsequent release of proinflammatory cytokines such as tumor necrosis factor-α, interleukin (IL)-1β, and IL-6 (3, 15, 16). This hyperinflammatory cascade disrupts alveolar–capillary integrity, increases vascular permeability, and amplifies the risk of acute respiratory distress syndrome (ARDS) and multiorgan dysfunction (17, 18).

Corticosteroids exert pleiotropic immunomodulatory effects that directly target these mechanisms. By binding to cytoplasmic glucocorticoid receptors, they translocate

to the nucleus and suppress transcription of proinflammatory genes while upregulating anti-inflammatory mediators (4, 15). Beyond cytokine suppression, corticosteroids stabilize endothelial and epithelial barriers, reduce leukocyte recruitment, and limit pulmonary edema (19–21). Preclinical studies suggest that corticosteroids may influence mitochondrial homeostasis and attenuate secondary tissue injury in non-infectious or *in vitro* injury models, although evidence in SCAP is lacking (22, 23). Moreover, observational and mechanistic studies have linked higher systemic inflammation (e.g., elevated IL-6, CRP) with worse outcomes in pneumonia (15–17), and randomized trials suggest that the largest clinical gains from adjunctive corticosteroids occur in patients with a pronounced inflammatory response (8, 13). These findings support the concept that modulation of the inflammatory milieu is the critical therapeutic target for corticosteroids in bacterial SCAP.

Beyond their anti-inflammatory actions, corticosteroids modulate both innate and adaptive immunity by promoting macrophage efferocytosis, enhancing clearance of apoptotic cells, and fostering a shift toward reparative immune phenotypes (24). They also upregulate anti-inflammatory mediators such as IL-10 and annexin A1, supporting resolution of inflammation and restoration of tissue homeostasis (25). In parallel, corticosteroids preserve endothelial integrity and regulate neutrophil trafficking, thereby limiting capillary leakage and alveolar injury. These actions indicate that corticosteroids not only suppress inflammation but also promote active disease resolution and tissue repair, providing a robust biological rationale for their use in bacterial SCAP characterized by severe systemic inflammation.

Taken together, these biological insights provide the rationale for investigating corticosteroids as an adjunctive therapy in bacterial SCAP. They also show that the net clinical effect is determined not only by the intensity of inflammation but also by the timing, dose, and duration of corticosteroid administration in clinical practice (11, 12).

## Evidence from recent randomized controlled trials

Over the last decade, several RCTs have evaluated adjunctive corticosteroids in patients with bacterial SCAP, but the results remain inconsistent (Table 1). The trial by Torres et al. (8) demonstrated that methylprednisolone reduced treatment failure in patients with severe pneumonia and elevated C-reactive protein (CRP >150 mg/L), suggesting benefit in those with a heightened inflammatory response. More recently, the CAPE-COD trial reported a significant reduction in 28-day mortality and increased organ support–free days with hydrocortisone in ICU patients with SCAP, reinforcing the potential utility of early corticosteroid therapy (6). Similarly, a multicenter subgroup analysis of the APROCCHSS trial reported a shorter time to clinical stability and fewer complications in patients with SCAP and septic shock who were randomized to hydrocortisone plus fludrocortisone vs. placebo (7).

**Table 1:** Summary of randomized controlled trials evaluating corticosteroids in patients with severe community-acquired pneumonia.

**Criterion**	**Torres (2015) *n* = 120 (8)**	**Meduri 2022 (ESCAPe) *n* = 584 (9)**	**Dequin 2023 (CAPE-COD) *n* = 800 (6)**	**Heming 2024 (APROCCHSS subgroup) *n* = 562 (7)**	**Angus 2025 (REMAP-CAP) *n* = 658 (10)**
Inclusion criteria	SCAP + CRP >150 mg/L	SCAP	SCAP + FiO_2_ ≥50% or invasive ventilation	Septic shock due to CAP	SCAP admitted to ICU (non-COVID)
Severity scores	•PSI 107 ± 38 vs. 110 ± 35	•PSI 125.6 ± 37.2 vs. 122.3 ± 34.4 •SOFA 6.68 ± 3 vs. 6.29 ± 2.85	•PSI 127 (102–153) vs. 130 (103–150) •SAPS II 37 (30–45) vs. 38 (31–47) •SOFA 4 (3–6) in both groups	•SAPS II 56 ± 19, •SOFA 12 ± 3	•APACHE II 18 (13–24) vs. 18 (13–22)
Inflammatory markers	CRP >150 mg/L	Not reported	CRP >150 mg/L in 70% participants	Not reported	Not reported
Pathogens identified	41% (26% bacteria, 15% virus)	43% (bacterial vs. viral not specified)	55% (46% bacteria, 9% virus)	72% (67% bacteria, 5% virus)	40% (30% bacteria, 10% virus/mixed)
Actual timing of steroids	Within 36 h of hospital admission	Median 40 h after hospital admission	Median 15 h after ICU admission	Within 24 h after septic shock onset	Median 8 h after ICU admission
Steroid types and doses	Methylprednisolone 0.5 mg/kg q12h × 5 days	Methylprednisolone 40 mg/day × 7 days, then taper (total 20 days)	Hydrocortisone 200 mg/day × 4 days + taper	Hydrocortisone 50 mg q6h + fludrocortisone 50 μg/day × 7 days	Hydrocortisone 50 mg q6h × 7 days
Primary outcome	↓ Treatment failure (13 vs. 31%)	No reduction in 60-day mortality (16 vs. 18%)	↓ 28-day mortality (6.2 vs. 11.9%)	↓ 90-day mortality (39 vs. 51%)	No reduction in 90-day mortality (15 vs. 9.8%)
Secondary outcomes	↓ Radiographic progression, ↓ CRP/IL-10	↓ Mechanical ventilation duration	↓ Intubation, ↓ vasopressor use, ↓ ICU stay	↓ ICU, hospital, 180-day mortality	↓ Vasopressor duration (~1 day)
Adverse events	Safe, no increase in complications	Safe, no increase in complications	↑ Hyperglycemia and insulin use	↑ Hyperglycemia	Safe, no increase in complications

SCAP, severe community-acquired pneumonia; CRP, C-reactive protein; IL, interleukin; APACHE, acute physiology and chronic health evaluation; SAPS, simplified acute physiology score; SOFA, sequential organ failure assessment score; PSI, pneumonia severity index; ICU: intensive care unit.

In contrast, other RCTs failed to demonstrate clear survival benefit. The ESCAPe trial found no significant effect of methylprednisolone on 60-day mortality or on any of the secondary outcomes (9). This study enrolled predominantly older male veterans, raising concerns about generalizability to broader ICU populations. Likewise, the REMAP-CAP adaptive platform trial screened more than 20,000 patients with pneumonia but ultimately randomized fewer than 1,000 and reporting neutral results for hydrocortisone with respect to mortality (10).

Some analyses have underscored the considerable heterogeneity across trials (11, 12). Key sources of variability include differences in baseline mortality rates, the proportion of patients with microbiological confirmation of bacterial etiology, the timing of corticosteroid initiation, and the specific regimens used. Notably, recent analyses suggest that specific corticosteroid types and dosing regimens may confer substantial benefit in selected high-risk subgroups, particularly those with elevated systemic inflammation (13, 14). However, broad application to all patients with bacterial SCAP has not consistently translated into improved survival (10). These findings highlight the importance of careful patient selection and standardized trial designs to clarify the role of corticosteroids in bacterial SCAP, with particular attention to phenotypes most likely to benefit, such as hyperinflammation, and to the determination of optimal treatment regimens.

## The challenge of heterogeneous etiologies in “bacterial” SCAP

One persistent obstacle in interpreting corticosteroid trials in SCAP is the uncertain microbiological landscape. Many studies included a substantial proportion of patients without microbiological confirmation or with mixed bacterial and viral infections (26, 27). In ESCAPe and REMAP-CAP, only slightly more than 40% of patients had an identified pathogen (with an even lower rate of confirmed bacterial infection; Table 1), while viral or mixed infections were not systematically excluded (9, 10). In contrast, the proportion of confirmed bacterial pathogens was considerably higher in CAPE-COD and in the APROCCHSS subgroup (46%−65%) (6, 7); importantly, the APROCCHSS subgroup included only patients with septic shock, 70% of participants in CAPE-COD and all patients in the trial by Torres et al. (8) had CRP >150 mg/L, collectively supporting the benefits of corticosteroids in a subgroup with a higher likelihood of bacterial infection and systemic hyperinflammation. This heterogeneity complicates treatment assessment, as corticosteroid responses may differ between purely bacterial pneumonia and mixed viral-bacterial cases. In viral pneumonias such as influenza A (H7N9) or MERS-CoV infection, corticosteroids have been associated with delayed viral clearance (28, 29), an increased risk of secondary infections, and higher mortality (30, 31), which may partly account for the inconsistent results of randomized trials, as variable inclusion of viral or mixed infections could obscure the true benefit of corticosteroids in bacterial SCAP.

Another source of variability is the distribution of pathogens. Pneumococcal pneumonia remains the prototypical bacterial SCAP, but Gram-negative bacilli and multidrug-resistant organisms are increasingly represented in ICU cohorts (32, 33). Different bacterial pathogens elicit distinct host immune responses. For example, pneumococcal infection is characterized by neutrophil-driven hyperinflammation, while Pseudomonas aeruginosa sepsis is more often associated with features of immune paralysis, and these differences may influence the net effect of corticosteroid therapy (34).

These challenges illustrate the risk of oversimplification when all cases of “bacterial SCAP” are grouped under a single therapeutic strategy. Without robust microbiological phenotyping and stratification, clinical trials risk diluting potential benefits in specific subgroups.

## The influence of inflammatory phenotypes

Accumulating evidence indicates that the host inflammatory phenotype is a critical determinant of response to corticosteroid therapy in SCAP. A recent individual patient data meta-analysis reported that adjunctive corticosteroids significantly reduced mortality only among patients with elevated systemic inflammation, defined by baseline CRP >204 mg/L, whereas those with lower CRP values derived no benefit (13). Consistent results were observed in critically ill patients, where treatment effects were strongly dependent on baseline inflammatory markers, with reductions in treatment failure and mortality confined to those with high inflammatory activity (35). These findings suggest that the benefit of corticosteroids is likely confined to hyperinflammatory phenotypes, whereas patients with hypoinflammatory profiles may even be harmed (36, 37). This biological divergence provides a plausible explanation for the heterogeneity of trial results, as unselected cohorts inevitably included both hyper- and hypoinflammatory patients (38–40).

Recent advances in acute respiratory distress syndrome (ARDS) research reinforce the dynamic nature of the inflammatory phenotype. Pensier et al. (41) identified hyperinflammatory and hypoinflammatory phenotypes that were not static, with many patients transitioning between states during the course of illness. Notably, corticosteroid benefit was observed primarily in those with persistent hyperinflammation, whereas patients who shifted toward hypoinflammation did not derive benefit. This observation may be directly relevant to bacterial SCAP, as a substantial proportion of patients enrolled in recent RCTs of corticosteroids met ARDS criteria according to the Berlin definition (42) or the updated global definition (43). These insights emphasize the importance of repeated assessment of inflammatory status in SCAP, not only to guide the initiation of corticosteroid therapy but also to support decisions on whether to maintain or withdraw therapy.

## Type, timing, dosing, tapering, and duration of corticosteroids

A major source of variability in trial outcomes is the heterogeneity of corticosteroid regimens. The five pivotal RCTs in bacterial SCAP adopted distinct approaches. Torres et al. (8) used intravenous methylprednisolone (0.5 mg/kg every 12 h for 5 days); the CAPE-COD trial administered hydrocortisone 200 mg/day by continuous infusion for 4 days, extended to a total of 8 or 14 days based on clinical improvement, with gradual tapering until ICU discharge (6); the ESCAPe trial evaluated a 20-day course of intravenous methylprednisolone, starting with a 40 mg bolus followed by 40 mg/day for 7 days, then tapered gradually to 20, 12, and 4 mg/day through day 20 (9); the APROCCHSS trial combined hydrocortisone 50 mg every 6 h for 7 days plus fludrocortisone (7); and REMAP-CAP used hydrocortisone 50 mg every 6 h for 7 days without systematic tapering (10). These differences in molecule, cumulative exposure, infusion schedule, and use of mineralocorticoid replacement highlight the lack of consensus on optimal steroid type, dose intensity, and treatment duration.

Mechanistic pharmacology provides a rationale for these divergent strategies. Hydrocortisone, with mineralocorticoid activity, supports vascular tone in septic shock and may be particularly advantageous when SCAP is complicated by vasodilatory collapse (6, 7, 44). In contrast, methylprednisolone achieves higher pulmonary tissue penetration (45), theoretically favoring alveolar inflammation. However, this pharmacologic advantage has not consistently translated into survival benefit in RCTs (8, 9), whereas hydrocortisone emerged as the only corticosteroid associated with mortality reduction in network meta-analysis (14).

As discussed in the section on inflammatory phenotypes, hyperinflammatory and hypoinflammatory states are not fixed and may evolve during the course of illness (41). This dynamic trajectory has important implications for corticosteroid tapering and timing. Tapering serves not only to prevent hypothalamic–pituitary–adrenal axis suppression and rebound cytokine release after abrupt discontinuation (46), but also to provide a planned reassessment point. In practice, tapering has two purposes including (1) to continue modulating a persistent hyperinflammatory state while avoiding abrupt withdrawal, and (2) to allow discontinuation if the patient has transitioned into a hypoinflammatory phenotype where further immunosuppression may be harmful (13, 41). Regarding timing, early initiation (often ≤ 24 h in trials) in patients with hyperinflammation may intercept the cytokine surge at its peak, whereas late initiation risks missing this window, potentially diminishing efficacy.

Importantly, a recent meta-analysis reported that adjunctive corticosteroids did not significantly increase serious adverse events such as gastrointestinal bleeding or secondary infections, although mild hyperglycemia and hypernatremia were more common (47). These metabolic disturbances are generally transient and manageable, but they underscore the need for close monitoring in critically ill patients. Corticosteroid exposure may increase the risk of neuromuscular weakness in critically ill patients with sepsis; however, this complication appears minimal and is outweighed by the survival benefits observed in bacterial SCAP (47, 48). When given at appropriate doses and with tapering, corticosteroids are safe but require close monitoring for metabolic and neuromuscular effects to balance benefit and harm.

## Clinical implications and future directions

The cumulative evidence from recent trials shows both the promise and the limitations of corticosteroid therapy in bacterial SCAP. Clinical translation requires moving beyond a “one-size-fits-all” paradigm toward approaches that account for heterogeneity in both inflammatory phenotype and therapeutic regimens.

First, stratification is essential. Microbiological distinctions and inflammatory phenotypes shape both the magnitude and the direction of steroid effects. Systematic pathogen identification and advanced diagnostics may help clarify which patients benefit or are harmed by corticosteroids. Evidence from sepsis and ARDS studies have integrated clinical features with biomarkers to define inflammatory profiles that identify patient subgroups most likely to benefit from corticosteroids (35, 37–41, 49–52). Similar investigations are warranted in patients with bacterial SCAP.

Second, regimen optimization remains a priority. Current data suggest that hydrocortisone, particularly when initiated early in hyperinflammatory states and tapered according to dynamic inflammatory phenotype, may provide the best balance of systemic and pulmonary effects (6, 7, 14). However, variation in dosing, duration, and tapering strategies across pivotal RCTs underscores the urgent need for standardized and adaptive protocols (6–10).

Finally, future research should embrace adaptive trial designs that integrate repeated inflammatory assessments, biomarker thresholds, and flexible treatment algorithms. Such designs could reconcile the conflicting results of past trials by targeting corticosteroid therapy to the right patients, at the right time, with the right dose. Ultimately, tailoring corticosteroid therapy according to host inflammatory status and pathogen profile may transform inconsistent trial results into consistent survival benefits for patients with severe bacterial pneumonia.

In conclusion, adjunctive corticosteroids hold promise in bacterial severe community-acquired pneumonia, but their effectiveness depends on underlying etiology, host inflammatory phenotype, and the treatment regimen applied. Future research should prioritize biomarker-guided strategies and standardized protocols to ensure corticosteroids are targeted to the right patients at the right time.

## References

[1] Jones BE Ramirez JA Oren E Soni NJ Sullivan LR Restrepo MI. Diagnosis and management of community-acquired pneumonia. Am J Respir Crit Care Med. (2025). 10.1164/rccm.202507-1692ST. [Epub ahead of print]. 40679934

[2] Martin-Loeches I Reyes LF Rodriguez A. Severe community-acquired pneumonia (sCAP): advances in management and future directions. Thorax. (2025) 80:565–75. 10.1136/thorax-2024-222296 40360263

[3] Rombauts A Abelenda-Alonso G Cuervo G Gudiol C Carratalà J. Role of the inflammatory response in community-acquired pneumonia: clinical implications. Expert Rev Anti Infect Ther. (2022) 20:1261–74. 10.1080/14787210.2021.1834848 33034228

[4] Rhen T Cidlowski JA. Antiinflammatory action of glucocorticoids–new mechanisms for old drugs. N Engl J Med. (2005) 353:1711–23. 10.1056/NEJMra050541 16236742

[5] Chaudhuri D Nei AM Rochwerg B Balk RA Asehnoune K Cadena R. 2024 focused update: guidelines on use of corticosteroids in sepsis, acute respiratory distress syndrome, and community-acquired pneumonia. Crit Care Med. (2024) 52:e219–33. 10.1097/CCM.0000000000006172 38240492

[6] Dequin PF Meziani F Quenot JP Kamel T Ricard JD Badie J. Hydrocortisone in severe community-acquired pneumonia. N Engl J Med. (2023) 388:1931–41. 10.1056/NEJMoa2215145 36942789

[7] Heming N Renault A Kuperminc E Brun-Buisson C Megarbane B Quenot JP. Hydrocortisone plus fludrocortisone for community acquired pneumonia-related septic shock: a subgroup analysis of the APROCCHSS phase 3 randomised trial. Lancet Respir Med. (2024) 12:366–74. 10.1016/S2213-2600(23)00430-7 38310918

[8] Torres A Sibila O Ferrer M Polverino E Menendez R Mensa J. Effect of corticosteroids on treatment failure among hospitalized patients with severe community-acquired pneumonia and high inflammatory response: a randomized clinical trial. JAMA. (2015) 313:677–86. 10.1001/jama.2015.88 25688779

[9] Meduri GU Shih MC Bridges L Martin TJ El-Solh A Seam N. Low-dose methylprednisolone treatment in critically ill patients with severe community-acquired pneumonia. Intensive Care Med. (2022) 48:1009–23. 10.1007/s00134-022-06684-3 35723686 PMC9208259

[10] Angus DC. Effect of hydrocortisone on mortality in patients with severe community-acquired pneumonia: the REMAP-CAP corticosteroid domain randomized clinical trial. Intensive Care Med. (2025) 51:665–80. 10.1007/s00134-025-07861-w 40261382 PMC12055926

[11] Ceccato A Russo A Barbeta E Oscanoa P Tiseo G Gabarrus A. Real-world corticosteroid use in severe pneumonia: a propensity-score-matched study. Crit care. (2021) 25:432. 10.1186/s13054-021-03840-x 34915895 PMC8674860

[12] Edland B Waterer GW. An overview of the recent trials of corticosteroids for severe community-acquired pneumonia. Expert Rev Respir Med. (2025) 19:925–33. 10.1080/17476348.2025.2513518 40483569

[13] Smit JM Van Der Zee PA Stoof SCM Van Genderen ME Snijders D Boersma WG. Predicting benefit from adjuvant therapy with corticosteroids in community-acquired pneumonia: a data-driven analysis of randomised trials. Lancet Respir Med. (2025) 13:221–33. 10.1016/S2213-2600(24)00405-3 39892408

[14] Zhu L Zeng J Li H Li K Chen X. Comparative effect of different corticosteroids in severe community-acquired pneumonia: a network meta-analysis. BMC Pulm Med. (2025) 25:210. 10.1186/s12890-025-03679-w 40307783 PMC12044815

[15] Kellum JA Kong L Fink MP Weissfeld LA Yealy DM Pinsky MR. Understanding the inflammatory cytokine response in pneumonia and sepsis: results of the genetic and inflammatory markers of sepsis (GenIMS) study. Arch Intern Med. (2007) 167:1655–63. 10.1001/archinte.167.15.1655 17698689 PMC4495652

[16] Remmelts HH Meijvis SC Biesma DH van Velzen-Blad H Voorn GP Grutters JC. Dexamethasone downregulates the systemic cytokine response in patients with community-acquired pneumonia. Clin Vaccine Immunol. (2012) 19:1532–8. 10.1128/CVI.00423-12 22855392 PMC3428389

[17] Bordon J Aliberti S Fernandez-Botran R Uriarte SM Rane MJ Duvvuri P. Understanding the roles of cytokines and neutrophil activity and neutrophil apoptosis in the protective versus deleterious inflammatory response in pneumonia. Int J Infect Dis. (2013) 17:e76–83. 10.1016/j.ijid.2012.06.006 23069683

[18] Meduri GU Annane D Chrousos GP Marik PE Sinclair SE. Activation and regulation of systemic inflammation in ARDS: rationale for prolonged glucocorticoid therapy. Chest. (2009) 136:1631–43. 10.1378/chest.08-2408 19801579

[19] Albers GJ Amouret A Ciupka K Montes-Cobos E Feldmann C Reichardt HM. Glucocorticoid nanoparticles show full therapeutic efficacy in a mouse model of acute lung injury and concomitantly reduce adverse effects. Int J Mol Sci. (2023) 24:16843. 10.3390/ijms242316843 38069173 PMC10705980

[20] Neyton LPA Patel RK Sarma A Ansel KM Christenson S Adkisson M. Distinct pulmonary and systemic effects of dexamethasone in severe COVID-19. Nat Commun. (2024) 15:5483. 10.1038/s41467-024-49756-2 38942804 PMC11213873

[21] Taenaka H Wick KD Sarma A Matsumoto S Ghale R Fang X. Biological effects of corticosteroids on pneumococcal pneumonia in Mice-translational significance. Crit Care. (2024) 28:185. 10.1186/s13054-024-04956-6 38807178 PMC11134653

[22] Du J McEwen B Manji HK. Glucocorticoid receptors modulate mitochondrial function: a novel mechanism for neuroprotection. Commun Integr Biol. (2009) 2:350–2. 10.4161/cib.2.4.8554 19721888 PMC2734045

[23] Park Y-J Heo J Kim Y Cho H Shim M Im K. Glucocorticoids alleviate particulate matter-induced COX-2 expression and mitochondrial dysfunction through the Bcl-2/GR complex in A549 cells. Sci Rep. (2023) 13:18884. 10.1038/s41598-023-46257-y 37919369 PMC10622527

[24] Meduri GU Annane D Confalonieri M Chrousos GP Rochwerg B Busby A. Pharmacological principles guiding prolonged glucocorticoid treatment in ARDS. Intensive Care Med. (2020) 46:2284–96. 10.1007/s00134-020-06289-8 33150472 PMC7641258

[25] Meduri GU Chrousos GP. General adaptation in critical illness: glucocorticoid receptor-alpha master regulator of homeostatic corrections. Front Endocrinol. (2020) 11:161. 10.3389/fendo.2020.00161 32390938 PMC7189617

[26] Cillóniz C Calabretta D Palomeque A Gabarrus A Ferrer M Marcos MÁ. Risk factors and outcomes associated with polymicrobial infection in community-acquired pneumonia. Arch Bronconeumol. (2025) 61:408–16. 10.1016/j.arbres.2025.01.001 39809696

[27] Liu Y-N Zhang Y-F Xu Q Qiu Y Lu Q-B Wang T. Infection and co-infection patterns of community-acquired pneumonia in patients of different ages in China from 2009 to 2020: a national surveillance study. Lancet Microbe. (2023) 4:e330–9. 10.1016/S2666-5247(23)00031-9 37001538 PMC12514336

[28] Arabi YM Mandourah Y Al-Hameed F Sindi AA Almekhlafi GA Hussein MA. Corticosteroid therapy for critically ill patients with middle east respiratory syndrome. Am J Respir Crit Care Med. (2018) 197:757–67. 10.1164/rccm.201706-1172OC 29161116

[29] Cao B Gao H Zhou B Deng X Hu C Deng C. Adjuvant corticosteroid treatment in adults with influenza A (H7N9) viral pneumonia. Crit Care Med. (2016) 44:e318–28. 10.1097/CCM.0000000000001616 26934144

[30] Moreno G Rodríguez A Reyes LF Gomez J Sole-Violan J Díaz E. Corticosteroid treatment in critically ill patients with severe influenza pneumonia: a propensity score matching study. Intensive Care Med. (2018) 44:1470–82. 10.1007/s00134-018-5332-4 30074052 PMC7095489

[31] Ni Y-N Chen G Sun J Liang B-M Liang Z-A. The effect of corticosteroids on mortality of patients with influenza pneumonia: a systematic review and meta-analysis. Crit Care. (2019) 23:99. 10.1186/s13054-019-2395-8 30917856 PMC6437920

[32] Cillóniz C Dominedò C Nicolini A Torres A. PES pathogens in severe community-acquired pneumonia. Microorganisms. (2019) 7:49. 10.3390/microorganisms7020049 30759805 PMC6406253

[33] Cillóniz C Dominedò C Torres A. Multidrug resistant gram-negative bacteria in community-acquired pneumonia. Crit Care. (2019) 23:79. 10.1186/s13054-019-2371-3 30850010 PMC6408800

[34] van der Poll T van de Veerdonk FL Scicluna BP Netea MG. The immunopathology of sepsis and potential therapeutic targets. Nat Rev Immunol. (2017) 17:407–20. 10.1038/nri.2017.36 28436424

[35] van Amstel RBE Bartek B Vlaar APJ Gay E van Vught LA Cremer OL. Temporal transitions of the hyperinflammatory and hypoinflammatory phenotypes in critical illness. Am J Respir Crit Care Med. (2025) 211:347–56. 10.1164/rccm.202406-1241OC 39642348 PMC11936145

[36] Koh MCY Lum LH Tambyah PA Ngiam JN. Inflammatory phenotypes and clinical outcomes amongst patients with presumed and confirmed *Pneumocystis jirovecii* pneumonia without underlying human immunodeficiency virus infection. Pathog Dis. (2025) 83:ftaf005. 10.1093/femspd/ftaf005 40275495 PMC12054482

[37] Antcliffe DB Burrell A Boyle AJ Gordon AC McAuley DF Silversides J. Sepsis subphenotypes, theragnostics and personalized sepsis care. Intensive Care Med. (2025) 51:756–68. 10.1007/s00134-025-07873-6 40163135 PMC12055953

[38] Sinha P Kerchberger VE Willmore A Chambers J Zhuo H Abbott J. Identifying molecular phenotypes in sepsis: an analysis of two prospective observational cohorts and secondary analysis of two randomised controlled trials. Lancet Respir Med. (2023) 11:965–74. 10.1016/S2213-2600(23)00237-0 37633303 PMC10841178

[39] Evrard B Sinha P Delucchi K Hendrickson CM Kangelaris KN Liu KD. Causes and attributable fraction of death from ARDS in inflammatory phenotypes of sepsis. Crit Care. (2024) 28:164. 10.1186/s13054-024-04943-x 38745253 PMC11092165

[40] DeMerle KM Kennedy JN Chang CH Delucchi K Huang DT Kravitz MS. Identification of a hyperinflammatory sepsis phenotype using protein biomarker and clinical data in the ProCESS randomized trial. Sci Rep. (2024) 14:6234. 10.1038/s41598-024-55667-5 38485953 PMC10940677

[41] Pensier J Fosset M Paschold BS von Wedel D Redaelli S Braeuer BLP. Temporal stability of phenotypes of acute respiratory distress syndrome: clinical implications for early corticosteroid therapy and mortality. Intensive Care Med. (2025). 10.1007/s00134-025-08089-4 40839098

[42] Ranieri VM Rubenfeld GD Thompson BT Ferguson ND Caldwell E Fan E. Acute respiratory distress syndrome: the Berlin definition. JAMA. (2012) 307:2526–33. 10.1001/jama.2012.5669 22797452

[43] Matthay MA Arabi Y Arroliga AC Bernard G Bersten AD Brochard LJ. A new global definition of acute respiratory distress syndrome. Am J Respir Crit Care Med. (2024) 209:37–47. 10.1164/rccm.202303-0558WS 37487152 PMC10870872

[44] Annane D Renault A Brun-Buisson C Megarbane B Quenot JP Siami S. Hydrocortisone plus fludrocortisone for adults with septic shock. N Engl J Med. (2018) 378:809–18. 10.1056/NEJMoa1705716 29490185

[45] Braude AC Rebuck AS. Prednisone and methylprednisolone disposition in the lung. Lancet. (1983) 2:995–7. 10.1016/S0140-6736(83)90981-9 6138595

[46] Williams DM. Clinical pharmacology of corticosteroids. Respir Care. (2018) 63:655–70. 10.4187/respcare.06314 29794202

[47] Chaudhuri D Israelian L Putowski Z Prakash J Pitre T Nei AM. Adverse effects related to corticosteroid use in sepsis, acute respiratory distress syndrome, and community-acquired pneumonia: a systematic review and meta-analysis. Crit Care Explor. (2024) 6:e1071. 10.1097/CCE.0000000000001071 38567382 PMC10986917

[48] Rochwerg B Oczkowski SJ Siemieniuk RAC Agoritsas T Belley-Cote E D'Aragon F. Corticosteroids in sepsis: an updated systematic review and meta-analysis. Crit Care Med. (2018) 46:1411–20. 10.1097/CCM.0000000000003262 29979221

[49] Reddy K O'Kane CM Antcliffe DB McDowell C Bradley PA Black L. Inflammatory phenotypes can be prospectively identified at the bedside in patients with the acute respiratory distress syndrome; results from a multicenter, prospective, observational cohort study. Am J Respir Crit Care Med. (2025) 211(Abstracts):A3130–A. 10.1164/ajrccm.2025.211.Abstracts.A3130

[50] Shankar-Hari M Calandra T Soares MP Bauer M Wiersinga WJ Prescott HC. Reframing sepsis immunobiology for translation: towards informative subtyping and targeted immunomodulatory therapies. Lancet Respir Med. (2024) 12:323–36. 10.1016/S2213-2600(23)00468-X 38408467 PMC11025021

[51] Sinha P Delucchi KL McAuley DF O'Kane CM Matthay MA Calfee CS. Development and validation of parsimonious algorithms to classify acute respiratory distress syndrome phenotypes: a secondary analysis of randomised controlled trials. Lancet Respir Med. (2020) 8:247–57. 10.1016/S2213-2600(19)30369-8 31948926 PMC7543720

[52] Sinha P Neyton L Sarma A Wu N Jones C Zhuo H. Molecular phenotypes of acute respiratory distress syndrome in the ROSE trial have differential outcomes and gene expression patterns that differ at baseline and longitudinally over time. Am J Respir Crit Care Med. (2024) 209:816–28. 10.1164/rccm.202308-1490OC 38345571 PMC10995566

